# Endothelial Effects in the Elderly: Fibroblast Regulation in Soft Tissue Healing

**DOI:** 10.1002/jcp.70099

**Published:** 2025-10-03

**Authors:** Martin Oberringer, Martina Jennewein, Monika Bubel, Silke Guthörl, Tim Pohlemann

**Affiliations:** ^1^ Department of Trauma‐, Hand‐ and Reconstructive Surgery Saarland University Homburg Germany

## Abstract

Two main influencing factors of human soft tissue healing are concomitant diseases and cellular senescence, both accumulating with increasing age. Due to the raising population of the elderly in western countries, it is essential to enhance the level of knowledge concerning the function of senescence in a granulation tissue during repair. The present study was intended to verify classic markers of senescence, like senescence‐associated ß‐galactosidase and the development of a senescence‐associated secretory phenotype among fibroblasts during emerging senescence. The application of an in vitro model using serial passaging as inducer of replicative senescence revealed specific differences of a non‐senescent and a pre‐senescent fibroblast phenotype in mono‐cultures, representing the basis for a detailed examination of the phenotypes in their interaction with microvascular endothelial cells in co‐cultures. The results deliver new insights into the age dependent process of tissue repair. Characteristics of pre‐senescent fibroblasts in terms of modified proliferation, cell morphology, cell cycle regulation, myofibroblastoid differentiation and cytokine release indicate a strong responsibility of this phenotype for the composition and function of a granulation tissue at different locations, including vascular sites. In its entirety, the results support the assumption, that a missing clearance of the senescence phenotype in late stages of tissue repair is one of the main reasons for healing failure.

## 1 Introduction

Subsequent to soft tissue trauma, regular healing relies on a well‐orchestrated cascade of events assuring tissue replacement (Diegelmann and Evans 2004). However, besides concomitant diseases, especially advanced age might impede healing, resulting in chronic wounds (Singh et al. 2017). While wound healing is quite uncomplicated in early life, formation of granulation tissue and wound closure delay with aging (Ding et al. 2021), and healing disorders are a widespread phenomenon in the elderly (Gould et al. 2015). Although established therapeutic treatment options are available, these are limited due to their difficult adaptation to the individual and unique character of a wound. Prognostic data of the population development in Germany show an increasing population of people older than 80 years from 6.1 million to date to more than 9 million in the year 2050 (Statistisches Bundesamt 2024), further indicating the urgent need of research in this field to provide advanced therapies.

Senescent cells, commonly designated as senescence associated secretory phenotype (SASP) (Coppé et al. 2008) are a regular part of older organisms. In soft tissue healing, the role of these cells was already discussed rather controversially. On one hand, they seem to impede healing (Shelton et al. 1999), mainly due to their pro‐inflammatory nature, but knowledge accumulates hinting to positive functions when they occur transiently during repair (Demaria et al. 2014; Davan‐Wetton et al. 2021). It is conceivable, that this role could especially be advantageous in early phases of healing, whereas chronicity due to an enhanced survival potential of a SASP provokes negative consequences, including tissue fibrosis and cancer (Wilkinson and Hardman 2020).

The proliferation phase of soft tissue healing is characterised by the arisal of a granulation tissue with a vigorous interaction of participating cells, such as tissue fibroblasts and endothelial cells (Rodrigues et al. 2019; Baum and Arpey 2005). Fibroblasts serve to assure proper tissue stabilisation and tension, provide extracellular matrix (ECM) proteins (Tracy et al. 2016) and are a source of α‐smooth muscle actin (α‐SMA) expressing myofibroblasts (MF), the latter of main importance during vascular development (Mayrand et al. 2012) and facilitating healing (Powell et al. 1999). Because the quality of granulation tissue is decisive for healing outcome and its development is delayed in the aged organism (Ding et al. 2021), it is obvious that functional changes occurring during aging of fibroblasts might have a massive impact. The interaction of fibroblasts with endothelial cells is of high importance for angiogenesis during healing (Hughes 2008). In a regular granulation tissue, endothelial cells of the microvasculature are crucial for the re‐establishment of a functional vascular network (Dulmovits and Herman 2012). Furthermore, chronic wounds are associated ‐or even caused to a certain extent‐ by vascular disease (Eming et al. 2014), so that the eminent role of endothelial cells becomes obvious.

Models allowing evidence on the impact of aging on soft tissue healing are limited. In vivo research by clinical trials remains difficult, due to regulatory hurdles and due to the unattainable homogeneity of the patient collectives to compare, which is caused by the broad individual spectrum of concomitant diseases. Here we modified an existing in vitro coculture model already applied in the context of soft tissue healing (Oberringer et al. 2007) to use fibroblasts with a difference in age, which were generated by serial passaging and replicative senescence (Hayflick and Moorhead 1961).

The present study was intended (i) to verify classic markers of senescence, like senescence‐associated ß‐galactosidase (SA‐β‐gal) (Dimri et al. 1995) and the development of a senescence‐associated secretory phenotype (SASP) (Coppé et al. 2008; Hernandez‐Segura et al. 2018) among fibroblasts during emerging senescence. (ii) Direct and indirect interaction of non‐senescent (non‐s) and pre‐senescent (pre‐s) fibroblasts with microvascular endothelial cells were examined in detail to attain a better understanding of those processes important for soft tissue healing in the elderly.

## 2 Material and Methods

### 2.1 Serial Cell Passaging

Primary normal human dermal fibroblasts (NHDF; PromoCell, Heidelberg, Germany) from a juvenile donor were cultured using fibroblast growth medium (FGM; Provitro, Berlin, Germany) without antibiotics, containing 10% foetal calf serum (FCS). From in vitro passage (P) 4 on, cells were passaged serially. Defined seeding numbers together with those measured during passaging enable the calculation of generation times and (cumulated) population doublings (Cristofalo et al. 1998). Cell morphology was documented by phase contrast microscopy. Surplus cells were stored in liquid nitrogen as cryoarchive. We ceased cell culture at P44, when population doubling did not occur within 20 days of culture.

Due to expected subtle changes of cell characteristics from one passage to the next, we defined three groups, with an arbitrary cut‐off for the group of ‘pre‐senescent (pre‐s) cultures’ at P34, which just was the passage, from which on the mean cell diameter remained larger than 20 µm. Another subdivision resulted in the groups of ‘non‐senescent (non‐s) cultures’ (P5–P17) and ‘cultures in transition’ (trans; P18–P33).

### 2.2 Characterisation of Fibroblasts by CASY Cell Counter

During serial passaging, a cell counter (CASY Model TT Cell Counter + Analyser System, Schärfe‐System GmbH, Reutlingen, Germany) was used to determine cell numbers, diameters and viabilities.

### 2.3 Characterisation of Fibroblasts by Senescence‐Associated ß‐Galactosidase

To quantitate SA‐ß‐gal of NHDF (*n* = 11), a slightly modified protocol of an assay introduced before was applied (Gary and Kindell 2005). In brief, SA‐ß‐gal quantities were determined as fluorescence intensity of 4‐methylumbelliferon measured by a microplate reader (Infinite M200, Tecan Deutschland GmbH, Crailsheim, Germany) and related to the total protein amount.

From the cryoarchive mono‐cultures of non‐s (P8–P9; *n* = 4) and pre‐s NHDF (P39–P42; *n* = 4) were prepared for a SA‐ß‐gal staining kit (Sigma, St. Louis, USA). 20 fields of view served to calculate the rate of SA‐ß‐gal positive cells.

### 2.4 Interaction of Fibroblasts With Endothelial Cells by Cell‐To‐Cell Contact

To verify characteristic responses of NHDF with different age to endothelial cells, we used a *direct* (cell‐to‐cell contact, *n* = 8 independent repetitions) and an *indirect* (conditioned medium, *n* = 8 independent repetitions) setup. Therefore, human dermal microvascular endothelial cells (HDMEC, PromoCell, Heidelberg, Germany) of two juvenile donors were cultured in Endothelial Cell Growth Medium MV (ECG‐MV; PromoCell), containing 5% FCS, and used in P5–P9.

For direct coculture non‐s (P8–P9; *n* = 4) and pre‐s (P39–P42; *n* = 4) NHDF of the cryoarchive were expanded, then (t‐24 h) seeded at 80 cells/mm^2^ together with HDMEC (160 cells/mm^2^) in coculture medium (CKM; one part FGM and one part ECG‐MV, 7.5% FCS,) on pretreated glass slides. For each repetition, additional mono‐cultures of NHDF (*n* = 4/*n* = 4) and HDMEC (*n* = 4/*n* = 4) were prepared in parallel. Cells were allowed to proliferate for another 24 h (day 0) and 72 h (day 2), before samples were fixed for immunocytochemical staining (Oberringer et al. 2007). Cell culture supernatants were collected for enzyme linked immunosorbent assay (ELISA).

#### 2.4.1 Immunocytochemical Staining

Immunostaining served to discriminate the cells into von Willebrand factor (vWF) positive HDMEC and vWF negative NHDF. Expression of α‐SMA indicated MF differentiation of NHDF. The rate of cells outside cell cycle phase G0 was determined by means of a Ki‐67 antibody. A list of first and secondary antibodies and dilutions is provided in Table S1 (Supporting Information). For nuclear counterstaining and coating a medium containing 4‘,6‐diamidino‐2‐phenylindole (DAPI; Vectashield, Vector Laboratories Burlingame, USA) was used.

#### 2.4.2 Quantitative Microscopy

During quantitative microscopy using an Axioskop 2 (Carl Zeiss Microscopy GmbH, Jena, Germany), equipped with Axiovision software (version 4.2.), 40 fields of view served to determine cell numbers and population densities, while the percentages of Ki‐67 and α‐SMA positive NHDF were determined by evaluating 20 fields of view.

### 2.5 Interaction of Fibroblasts With Endothelial Cells by Conditioned Medium

Indirect co‐culture served to verify paracrine effects transferred by HDMEC conditioned medium to non‐s (P8–P10; *n* = 4) and pre‐s NHDF (P41–P43; *n* = 4). At t‐48 h mono‐cultures of NHDF (60 cells/mm^2^) and HDMEC (160 cells/mm^2^) were seeded in the referring media. Subsequent to a medium change to CKM at t‐24 h, HDMEC produced conditioned medium over a period of 24 h; non‐conditioned CKM served as control. NHDF were exposed to conditioned and non‐conditioned CKM for another 48 h, followed by cell counting and isolation of RNA. Cell culture supernatants were stored for ELISA initially and after 48 h (in addition non‐conditioned and conditioned CKM samples without NHDF contact were collected).

#### 2.5.1 Quantitative Real‐Time Polymerase Chain Reaction

Total RNA from non‐s (*n *= 4) and pre‐s NHDF (*n* = 4), either cultured in non‐conditioned or conditioned medium, served for reverse transcription and gene expression analysis, as described earlier (Bachmann et al. 2020). To obtain a substantial basis for the relation of gene expression data with those from the other analyses of the study, a broad range of senescence associated genes (Table S2, Supporting Information) was included, representing a large spectrum of different cell functions: P16, P21, P38 and P53 are coding for proteins important for cell cycle regulation, while VIM is coding for vimentin, a major cytoskeletal filament protein. Genes coding for proteins of the ECM with high relevance for tissue healing like fibronectin and collagens are represented by FN1, COL1A1 and COL3A1. In addition, genes from the group of cytokines and growth factors, namely interleukin (IL)‐6, IL‐8, monocyte chemoattractant protein‐1 (MCP‐1) and vascular endothealial growth factor (VEGF) were analysed in the context of inflammation. The reference gene was GAPDH.

### 2.6 Enzyme‑Linked Immunosorbent Assay (ELISA)

In cell culture supernatants of both the direct and the indirect setup concentrations of IL‐6 and MCP‐1 were analysed by Quantikine ELISA Human kits (R&D systems, Minneapolis, USA). A microplate reader was used to measure absorbance. Cytokine concentrations were calculated by Magellan Software (V 7.2., Tecan).

### 2.7 Data Groups and Statistic Evaluation

To identify significant differences between three groups we used one way analysis of variance (ANOVA) and ANOVA on ranks in the case of non‐given normality. For differences between two groups we applied student's t‐test (Mann‐Whitney‐Rank‐Sum test in the case of non‐given normality, Welch's t‐test in the case of non‐given equal variance). Values in the graphics are given as individual data points with means and standard deviation (SD).

## 3 Results

### 3.1 Pre‐senescence of Fibroblasts by Serial Passaging

**Figure 1:**
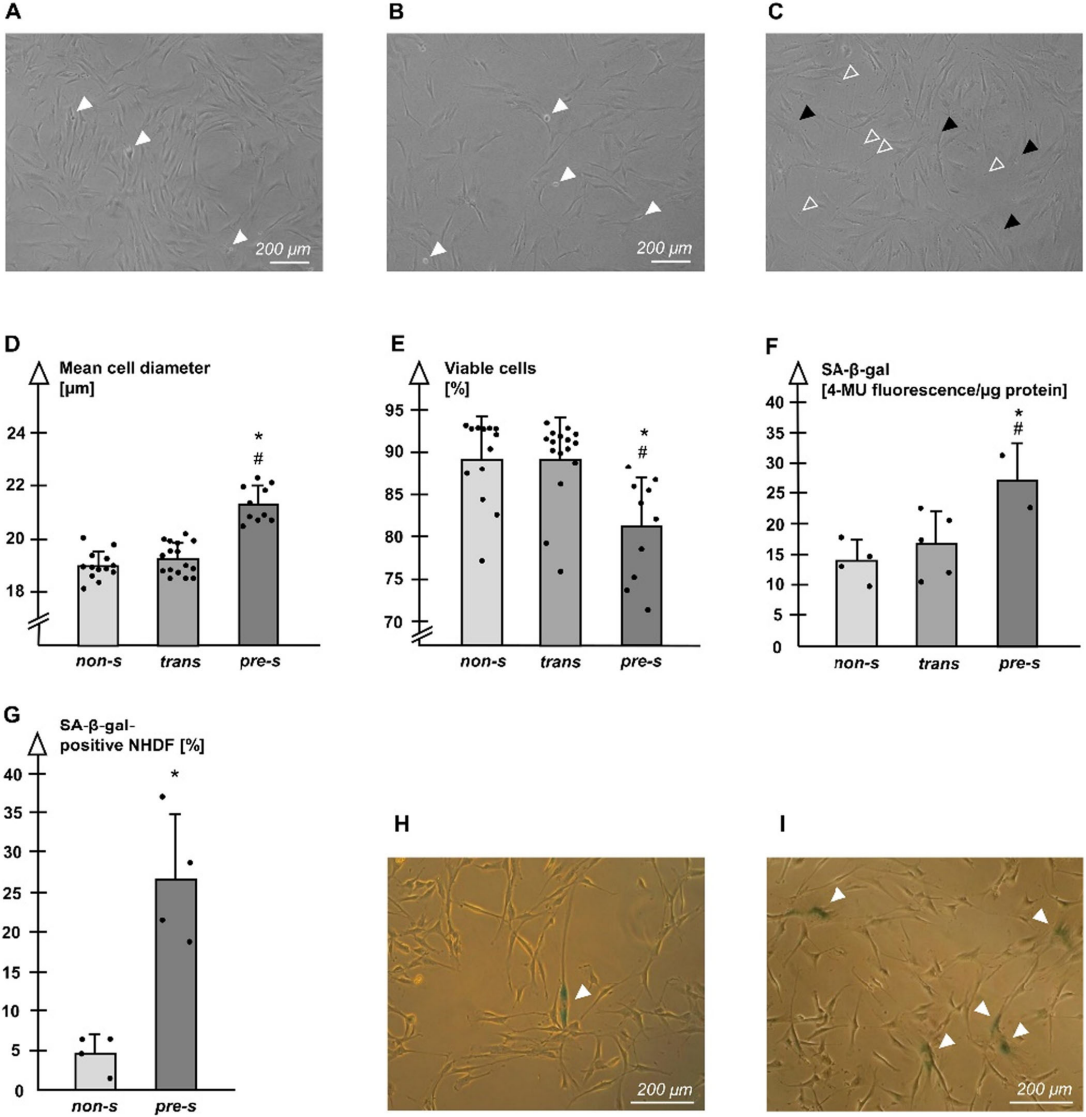
(A) NHDF in non‐senescent (non‐s) cultures exhibiting spindle shaped morphology and mitoses (arrowheads). (B) NHDF of cultures in transition (trans) still showing multiple mitoses (arrowheads). (C) NHDF in pre‐senescent (pre‐s) cultures with increased surface area (dark arrowheads) and cellular leftovers (open arrowheads). (D) NHDF in pre‐s cultures are larger compared to cells of trans cultures and to those in non‐s cultures. (E) Pre‐s cultures show a weaker viability. (D/E) Non‐s: *n* = 13; trans: *n* = 16; pre‐s: *n* = 10. (F) SA‐β‐gal is increased in pre‐s cultures (non‐s: *n* = 4, trans: *n* = 5, pre‐s: *n* = 2), *p* < 0.05, *versus non‐s; ^#^versus trans. (G) Pre‐s cultures from the cryoarchive still show an increased rate of SA‐β‐gal positive NHDF compared to non‐s cultures; *n* = 4, *p* < 0.05, *versus non‐s. (H,I) Phase contrast microphotographs of SA‐β‐gal positive NHDF (arrowheads) in a non‐s culture (H) and in a pre‐s culture (I).

Phase contrast microscopy of NHDF revealed the typical spindle shaped morphology in early passages (Figure 1A,B), whereas more and more cells with a larger cell surface occurred with progressive passaging (Figure 1C). In late passages cellular leftovers on the culture surface were present frequently (Figure 1C). Proliferation in terms of population doublings per passage was declining from 1.86 in P5 to 0.40 in P43, where NHDF finally had undergone a total of 44 cumulated population doublings. Mean cell diameters determined by CASY impressively increased over passaging (Figure 1D), while the mean viability was decreasing (Figure 1E). SA‐β‐gal values were increased in the group of pre‐s cultures (Figure 1F). These data clearly validate the generation of a pre‐s NHDF phenotype by serial passaging. The classic marker SA‐β‐gal was preserved during cryostorage, indicated by a higher rate of positive NHDF in pre‐s cultures (Figure 1G–I).

### 3.2 Proliferation of Pre‐S NHDF is Weaker, but not Restricted by HDMEC

**Figure 2:**
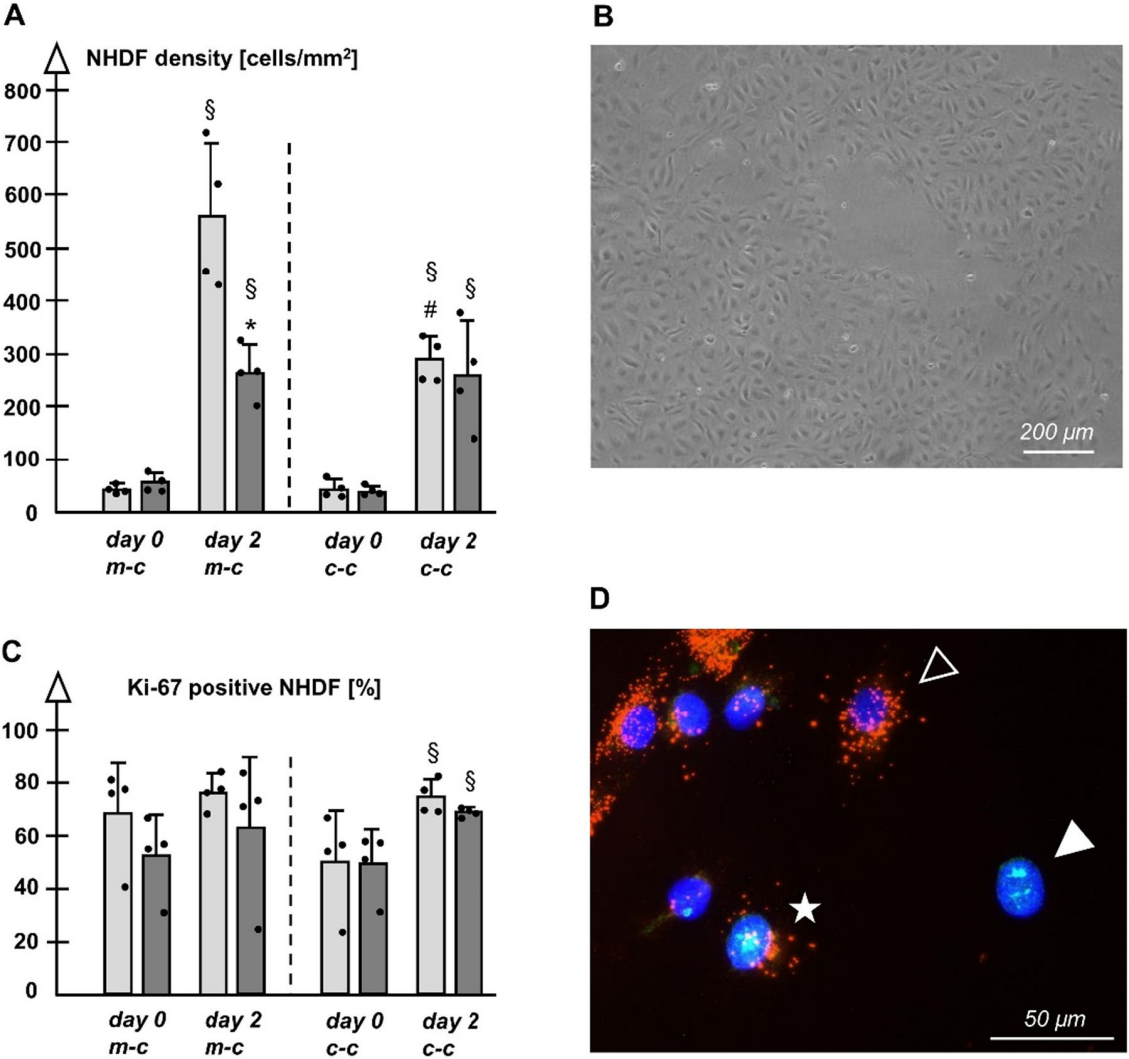
(A) Proliferation (cell density) of non‐s (light columns) and pre‐s NHDF (dark columns) in mono‐culture (m‐c) and in co‐culture (c‐c). (B) Representative morphology of HDMEC in mono‐culture revealed by phase contrast microscopy. (C) Proliferation (rate of Ki‐67 positive cells) of non‐s and pre‐s NHDF in m‐c and in c‐c. (A/C) *n* = 4 each, *p* < 0.05, ^**§**^versus day 0, *versus non‐s at the same time point, ^**#**^versus m‐c. (D) Fluorescence microphotograph of co‐cultured NHDF and HDMEC, subsequent to nuclear staining of Ki‐67 and vWF staining of HDMEC, showing a Ki‐67 negative (empty arrowhead, Cy3^TM^ [red]) and positive (asterisk, Cy3^TM^ [red], Alexa Fluor 488 [green]) HDMEC and a Ki‐67 positive NHDF (white arrowhead, Alexa Fluor 488 [green]); cell nuclei are stained with DAPI (blue).

In mono‐culture, non‐s NHDF density reflecting proliferation during 2 days was increased, while a significantly weaker proliferation was detected for pre‐s NHDF (Figure 2A). Intense proliferation of non‐s NHDF observed in mono‐culture was restricted by HDMEC (Figure 2B) in co‐culture, whereas such an effect was not given for pre‐s NHDF (Figure 2A).

Proliferation data in terms of the rate of cycling cells, which are not in the resting stage G0 of the cell cycle, indicated no difference between non‐s and pre‐s NHDF (Figure 2C,D): In the mono‐cultures, there was a quite similar rate of Ki‐67 positive NHDF on day 0 and on day 2. However, the presence of HDMEC in co‐cultures initially dampened the rate of cycling NHDF by trend at day 0. Re‐initiation of the cell cycle occurred on day 2.

### 3.3 The Presence of Myofibroblasts Is Reduced Among Pre‐S NHDF and is not Triggered by HDMEC

**Figure 3:**
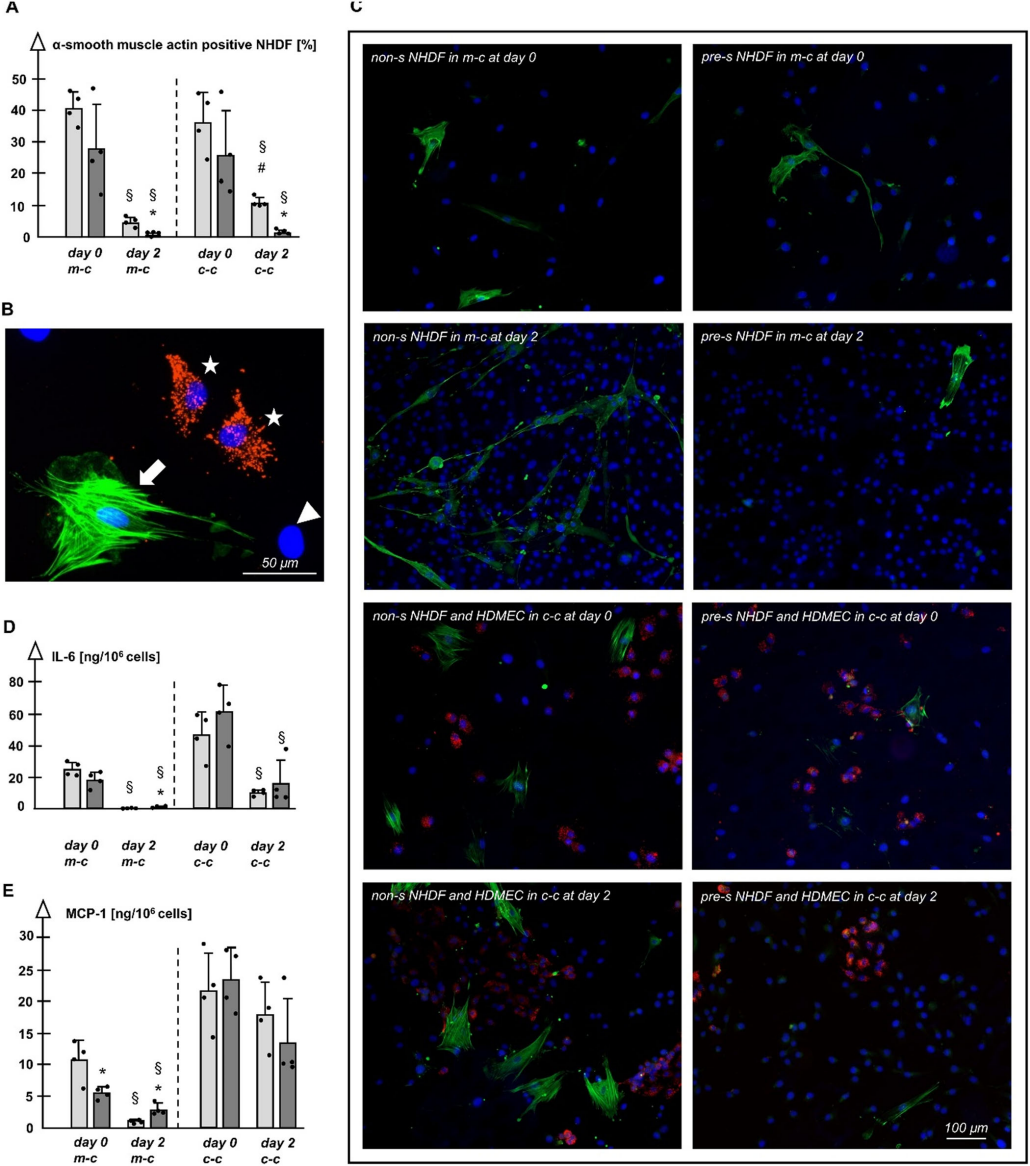
(A) Rate of myofibroblasts in non‐s (light columns) and pre‐s (dark columns) mono‐culture (m‐c) and co‐culture (c‐c); *n* = 4 each, *p* < 0.05, ^§^versus day 0, *versus non‐s NHDF at the same time point, ^#^versus m‐c. (B) Fluorescence microphotograph of a co‐culture; HDMEC with typical vWF‐expression (asterisks, Cy3^TM^ [red]), an α‐SMA negative NHDF (arrowhead, blue nucleus [DAPI]) and an α‐SMA positive myofibroblastoid NHDF (arrow, Alexa Fluor 488 [green]); cell nuclei are stained with DAPI (blue). (C) Representative microphotographs of pre‐s and non‐s NHDF in m‐c and c‐c with HDMEC at the different time points (HDMEC: Cy3^TM^ [red], α‐SMA negative NHDF: blue nucleus [DAPI], α‐SMA positive myofibroblastoid NHDF: Alexa Fluor 488 [green]). (D/E) IL‐6/MCP‐1 in cell culture supernatants of non‐s and pre‐s NHDF in m‐c and in c‐c with HDMEC; *n* = 4 each, *p* < 0.05, ^§^versus day 0, *versus non‐s NHDF at the same time point.

In general, α‐SMA positive NHDF reached maximum values at day 0 and decreased over time (Figure 3A–C). Interestingly, pre‐s NHDF showed lower rates of MF compared to non‐s cultures at day 2. The rate of MF could by triggered by presence of HDMEC in co‐culture in the case of non‐s NHDF on day 2, but not among pre‐s NHDF.

### 3.4 The Cytokine Release of Pre‐S NHDF Indicates the Development of a SASP

IL‐6 levels of mono‐ and co‐cultures decreased up to day 2 (Figure 3D). In co‐culture with HDMEC all measured values were in a higher range. In this context, additionally prepared HDMEC mono‐cultures only weakly released IL‐6 (0.91 ng/10e6 cells at day 0 and 1.24 ng/10e6 cells at day 2) and thus seem not to play a major role for total IL‐6 release in the co‐cultures. Pre‐s NHDF showed a higher IL‐6 value compared to non‐s NHDF in the mono‐culture on day 2. This is a clear hint towards the development of a SASP in pre‐s cultures.

In the mono‐culture, MCP‐1 levels decreased over time up to day 2 (Figure 3E). Compared to non‐s NHDF, pre‐s NHDF released more MCP‐1 at day 2, indicating the development of a SASP. Lower levels of MCP‐1 among pre‐s NHDF at the early time point might hint to a lower stress level during the initial cell adhesion process. Again, all measured values were in a higher range in co‐cultures with HDMEC. MCP‐1 release of HDMEC in mono‐cultures (29.09 ng/10e6 cells at day and 55.59 ng/10e6 cells at day 2) implies, that in co‐cultures most MCP‐1 is derived from HDMEC and not from NHDF.

### 3.5 Gene Expression Profiles Indicate Distinct Regulation in Non‐S and Pre‐S NHDF

**Figure 4:**
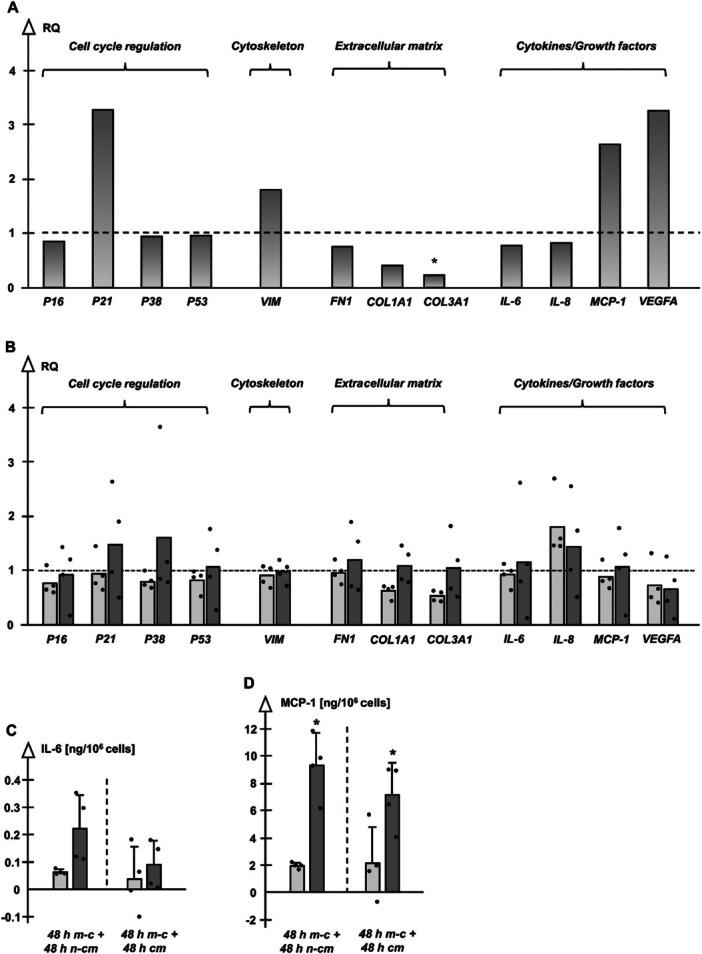
(A) Gene expression (relative quantification (RQ) = 2^−ΔΔCt^) of pre‐s vs. non‐s NHDF cultures; RQ calculated from mean ΔCt values (*n* = 4), *p* < 0.05, *pre‐s versus non‐s NHDF (ΔCt values). (B) Gene expression of non‐s (light columns) and pre‐s cultures (dark columns) in HDMEC‐conditioned medium compared to cultures in nonconditioned medium (*n* = 4, mean RQ). (C/D) Release of IL‐6/MCP‐1 in cell culture supernatants of non‐s (light columns) and pre‐s (dark columns) NHDF subsequent to culturing first in mono‐culture medium (48 h m‐c) and then in nonconditioned (+48 h n‐cm) or HDMEC‐conditioned (+48 h cm) medium; *n* = 4 each, *p* < 0.05, *versus non‐s NHDF at the same time point; values for cytokines delivered by HDMEC cm were subtracted before to display only cytokine values released by NHDF.

The comparison of gene expression of non‐s and pre‐s NHDF resulted in obvious trends: In case of growth in non‐conditioned medium ‐with absent influence of HDMEC‐ pre‐s NHDF showed increased expression of cell cycle regulator P21, when compared to non‐s NHDF (Figure 4A), while P16, P38 and P53 were in a similar range. VIM was up‐regulated in pre‐s NHDF, whereas all ECM associated genes ‐FN1, COL1A1 and COL3A1‐ were down‐regulated. Among the cytokine/growth factor genes, especially MCP‐1 and VEGFA showed higher expression in pre‐s NHDF. IL‐6 and IL‐8 ranged at a similar expression level.

### 3.6 Non‐S and Pre‐S NHDF Show Different Responses to HDMEC Conditioned Medium

Gene expression changes in response to conditioned media ranged at a low level in the present setup. However, the interpretation of the observed trends, whether a certain gene is up‐ or down‐regulated, serve to indicate a general difference in gene regulation between non‐s and pre‐s NHDF.

Conditioned medium of HDMEC stabilised P21 and P38 in pre‐s NHDF (Figure 4B). There was no effect on gene expression of VIM. Among pre‐s cultures genes of the ECM were stable, whereas non‐s NHDF showed down‐regulation of COL1A1 and COL3A1. Cytokine/growth factor genes were regulated similarly in both NHDF groups: IL‐8 expression was triggered and VEGFA expression was dampened, while there was no clear change of IL‐6 and MCP‐1. However, the influence of HDMEC on the deflection direction of IL‐6 and MCP‐1 gene expression was different: While non‐s NHDF responded with a slight down‐regulation of IL‐6 and MCP‐1 genes, pre‐s NHDF showed a more heterogeneous response resulting in a slight up‐regulation of IL‐6 and MCP‐1. Therefore, it was reasonable to obtain a more detailed view in terms of the release of the referring proteins by ELISA.

### 3.7 The Cytokine Release of Non‐S and Pre‐S NHDF is not Modified by HDMEC

The release of IL‐6 and MCP‐1 proteins was not clearly influenced by HDMEC‐conditioned medium in both NHDF groups. However, these additional data support the day 2 data derived from the direct interaction setup, where IL‐6 and MCP‐1 ranged at a higher level in pre‐s NHDF. Especially higher MCP‐1 values of pre‐s NHDF again indicate the development of a SASP (Figure 4C,D).

## 4 Discussion

NHDF population doublings decreased continuously with progressing passages, as expected. Pre‐s cultures showed characteristic signs of emerging senescence, such as a viability decrease, increased SA‐ß‐gal, weak proliferation due to up‐regulation of P21, as well as increased cell size. As large cell size is a typical sign of senescence (Hernandez‐Segura et al. 2018), it is obvious, that these cells might no longer contribute to proliferation, because of an intensive cell cycle checkpoint control and impaired cytoskeletal functionality (Rebehn et al. 2023).

Pre‐s NHDF showed leftovers on the culture surface, resembling to what is termed ‘traces’ in the literature (Fuhr et al. 1998). Here, the ‘traces’ might reflect a certain loss of the general cell body flexibility during cell migration and protrusion retraction, indicating an increased adherence potential of pre‐s NHDF compared to non‐s NHDF. In this context, a tendential up‐regulation of vimentin, a known feature of enlarged senescent fibroblasts (Nishio et al. 2001), was also detected among pre‐s NHDF, again indicating a modified cytoskeletal composition affecting adhesion behaviour.

**Figure 5:**
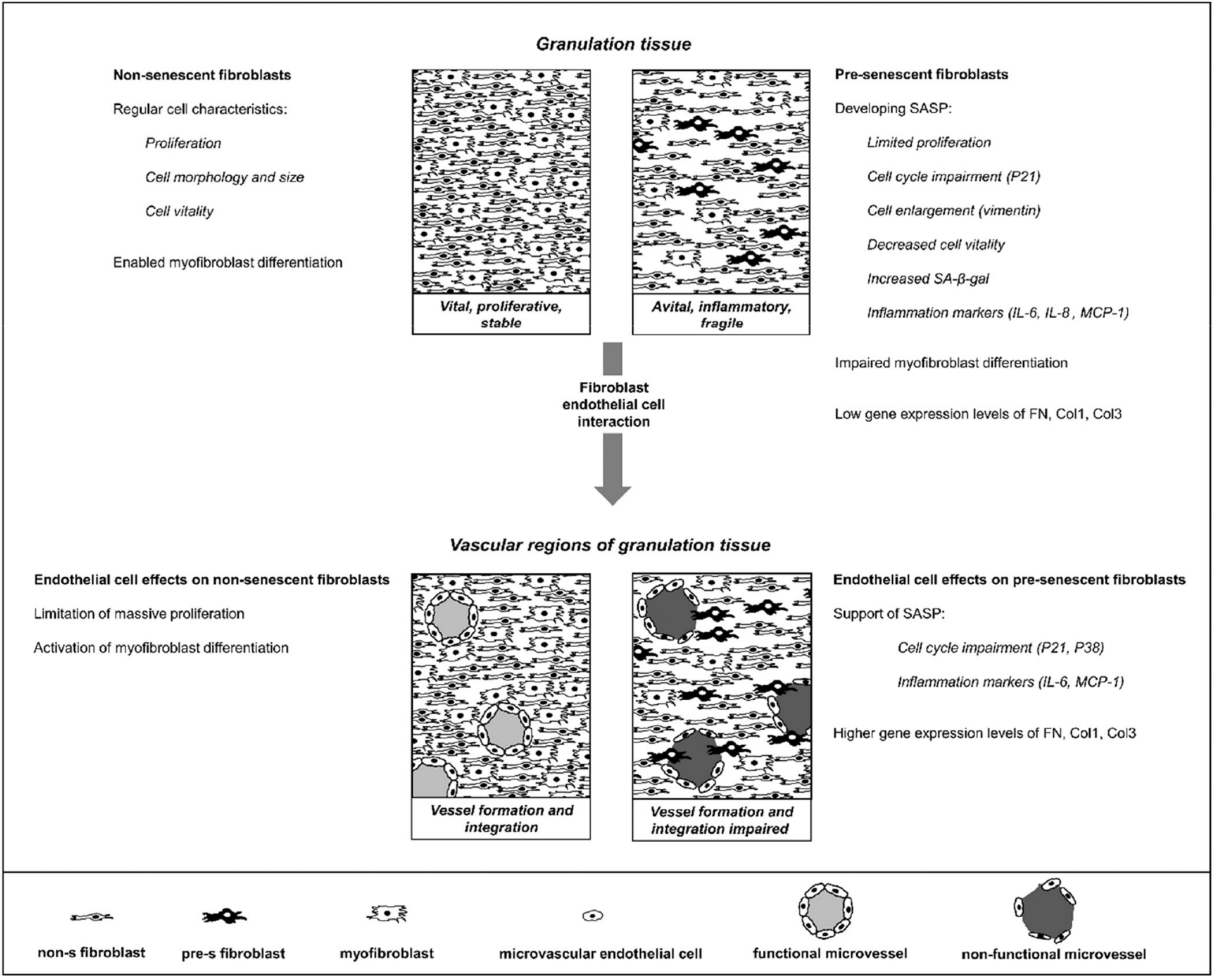
Specific features of non‐senescent and pre‐senescent fibroblasts determine granulation tissue quality and healing outcome (details see text).

Translating these characteristics into the in vivo situation of a developing granulation tissue indicates, that the presence of a pre‐s phenotype is disadvantageous and provokes an avital tissue (Figure 5). In contrast, a functional granulation tissue develops on the basis of non‐s NHDF by regular cell proliferation, viability, morphology and size. SASP characteristics among pre‐s, as indicated by the inflammation markers IL‐6 and MCP‐1 already present in mono‐cultures in the direct interaction model, leads to the assumption, that pre‐s NHDF have a rather severe paracrine influence on the microenvironment in a granulation tissue. It is thus obvious, that emerging and persisting pro‐inflammatory SASP might impede healing and support chronic wounds.

Furthermore, we conclude from our data, that impaired differentiation of pre‐s NHDF to MF could be a main cause of tissue destabilization leading to a fragile granulation tissue in the elderly. MF are responsible for tissue contraction (Gabbiani et al. 1972) and deliver important ECM proteins during regular healing (Bagalad et al. 2017), so that a lack of MF directly explains a missing stabilisation. Gene expression of pre‐s NHDF supports this assumption by low levels of FN as well as COL1A1 and COL3A1. Down‐regulation of COL1A1 and COL3A1 was shown before with respect to aged fibroblasts (Brun et al. 2016).

As the re‐establishment of a functional microvascular system by vessel repair, angiogenesis and angiointegration is essential for the performance of a granulation tissue, we focused onto those events occurring in vascular regions by bringing HDMEC into direct and indirect contact to non‐s and pre‐s NHDF. HDMEC had a different influence on non‐s and pre‐s NHDF: In vicinity to HDMEC non‐s NHDF were unable to reach such a proliferation, which was present in the referring mono‐cultures. However, because the amount of resting cells in cell cycle phase G0 was similar for non‐s and pre‐s NHDF, this proliferation dampening was likely not due to a G0 arrest.

Transferring these results to the in vivo situation means, that the capability of microvascular endothelial cells to restrict excessive fibroblast proliferation in a granulation tissue might reflect a mechanism, by which the total cell mass, mainly delivered by fibroblasts, can be limited. This seems to be important during the transition of the proliferation phase into the remodelling phase of soft tissue healing to assure functional integration of newly formed and repaired vessels into the maturing tissue. It is also worth mentioning in this context, that Mayrand et al. 2012 already described ‘that a strong increase in cell number can inhibit capillary formation.’

Such a limiting effect of HDMEC seems not to be necessary in the case of pre‐s NHDF, because pre‐s NHDF do not show strong proliferation in contact to HDMEC, mainly due to the maintenance of high P21 levels. P21 up‐regulation can lead to a cell cycle impairment by controlling both checkpoints, at G1/S and at G2/M (Hoeferlin et al. 2011; Al Bitar and Gali‐Muhtasib 2019) and cause a protection of senescent cells from death (Yosef et al. 2017). Interestingly enough, pre‐s NHDF additionally respond to HDMEC by up‐regulation of P38. In consequence, and with respect to the in vivo situation, cell cycle regulation at G1/S and G2/M by P38 (Thornton and Rincon 2009) might additionally support impaired proliferation.

Especially stable expression of MCP‐1 and IL‐6 among pre‐NHDF by conditioned medium indicates a massive impact of the microvasculature in the elderly in vivo. Besides the presence of pro‐inflammatory cytokines, down‐regulation of VEGFA and re‐enforcing FN, COL1A1 and COL3A1 gene expression of aged fibroblasts located in vascular vicinity might hinder microvessel formation and their proper integration into the tissue, leading to impaired healing.

In addition, the permanent lack of MF among pre‐s NHDF could account for disturbed angiogenesis and re‐establishment of vascularisation, as MF ‘provide a favourable environment for vascular development’ (Mayrand et al. 2012). In our model, MF differentiation was triggered by HDMEC in the case of non‐s NHDF. In contrast, pre‐s NHDF did not respond to HDMEC in such a way, indicating a disregulated cell communication, probably supported by lost self‐activating properties of MF occurring with age (Brun et al. 2016).

## 5 Conclusion

The characteristic behaviour of NHDF in this model supports the current understanding of the beneficial and detrimental roles of cellular senescence in tissue repair and delivers new results and perspectives on the delicate interaction with endothelial cells. The results have a high relevance for the interpretation of the in vivo processes occurring in a granulation tissue, with a special focus on vascular regions. Among non‐s NHDF a proliferation dampening by HDMEC was noticed, wheras pre‐s NHDF generally showed a weaker proliferation, most likely caused by maintenance of P21 and P38 levels. Besides the appearance of ‘traces’ in pre‐s NHDF cultures, indicating a certain loss of cell flexibility, SASP characteristics in terms of limited viability, increased cell size and presence of inflammation markers were detected. The differentiation rates of pre‐s NHDF to MF were low and could not be triggered by HDMEC. Therefore, severe functional deficiency with respect to tissue stabilisation, angiogenesis and the re‐establishment of vascularisation is assumed for pre‐s NHDF.

With respect to the persistence of fibroblasts with a senescent phenotype in later healing stages, the phenomenon of an inefficient clearance of these cells might in part be caused by their local interaction with endothelial cells at vascular sites. This aspect should be one integral part of those future studies on soft tissue healing designed for the elderly.

## Author Contributions

All authors participated in the conceptual design of the study. Martina Jennewein, Silke Guthörl and Martin Oberringer performed NHDF cell culture as well as CASY measurements and data collection. The 4‐MU‐assay was performed by Silke Guthörl and SA‐ß‐gal staining by Monika Bubel. Martin Oberringer wrote the initial manuscript draft. All authors agreed with the final version.

## Conflicts of Interest

The authors declare no conflicts of interest.

## Supporting information


**Table S1:** List of antibodies and dilutions applied in immunocytochemical staining.

**Table S2:** List of gene names, abbreviations and assay ID of TaqMan^TM^ FAM/MGB probes applied in quantitative real‐time polymerase chain reaction.

## Data Availability

The data that support the findings of this study are available from the corresponding author upon reasonable request.
